# Napping during major depressive episodes identifies a distinct hypersomnolence phenotype

**DOI:** 10.1093/sleepadvances/zpag097

**Published:** 2026-09-18

**Authors:** Pierre Alexis Geoffroy, Célia Heiligenstein, Sibylle Mauries, Justin Ulm, Fériel Zehani, Chantal Henry, Michel Lejoyeux, Patrice Bourgin, Julia Maruani

**Affiliations:** Département de psychiatrie et d'addictologie, AP-HP, GHU Paris Nord, DMU Neurosciences, Hôpital Bichat–Claude Bernard, Paris F-75018, France; Université Paris Cité, NeuroDiderot, Inserm, Paris F-75019, France; Centre ChronoS, GHU Paris–Psychiatry & Neurosciences, Paris 75014, France; CNRS UPR 3212, Institute for Cellular and Integrative Neurosciences, Strasbourg University, F-67000, France; Hôpitaux Universitaires de Strasbourg, Service de Pédopsychiatrie de l’Enfant et de l’Adolescent Strasbourg; CIRCSom (International Research Center for ChronoSomnology) & Sleep Disorders Center, Strasbourg University Hospital, Strasbourg, F-67000, France; Département de psychiatrie et d'addictologie, AP-HP, GHU Paris Nord, DMU Neurosciences, Hôpital Bichat–Claude Bernard, Paris F-75018, France; Centre ChronoS, GHU Paris–Psychiatry & Neurosciences, Paris 75014, France; Emergency Department, Percy military teaching hospital, Clamart 92140, France; Département de psychiatrie et d'addictologie, AP-HP, GHU Paris Nord, DMU Neurosciences, Hôpital Bichat–Claude Bernard, Paris F-75018, France; Centre ChronoS, GHU Paris–Psychiatry & Neurosciences, Paris 75014, France; Université Paris Cité, NeuroDiderot, Inserm, Paris F-75019, France; Service Hospitalo Unievrsitaire (SHU), GHU Paris–Psychiatry & Neurosciences, Paris 75014, France; Département de psychiatrie et d'addictologie, AP-HP, GHU Paris Nord, DMU Neurosciences, Hôpital Bichat–Claude Bernard, Paris F-75018, France; Université Paris Cité, NeuroDiderot, Inserm, Paris F-75019, France; Centre ChronoS, GHU Paris–Psychiatry & Neurosciences, Paris 75014, France; CNRS UPR 3212, Institute for Cellular and Integrative Neurosciences, Strasbourg University, F-67000, France; Hôpitaux Universitaires de Strasbourg, Service de Pédopsychiatrie de l’Enfant et de l’Adolescent Strasbourg; CIRCSom (International Research Center for ChronoSomnology) & Sleep Disorders Center, Strasbourg University Hospital, Strasbourg, F-67000, France; Département de psychiatrie et d'addictologie, AP-HP, GHU Paris Nord, DMU Neurosciences, Hôpital Bichat–Claude Bernard, Paris F-75018, France; Université Paris Cité, NeuroDiderot, Inserm, Paris F-75019, France; Centre ChronoS, GHU Paris–Psychiatry & Neurosciences, Paris 75014, France

**Keywords:** nap, depression, unipolar disorders, bipolar disorders, excessive daytime sleepiness, biological clock, sleep–wake disorder

## Abstract

**Study Objectives:**

Prior studies have linked depression with napping, though findings remain inconsistent. Given that major depressive episodes (MDE) commonly involve sleep–wake and circadian disturbances, we hypothesized that daytime Napping during MDE reflects more pronounced sleep/circadian disruptions and a distinct clinical profile.

**Materials and Methods:**

We compared MDE patients with and without napping (MDE-NAP vs MDE), as well as unipolar and bipolar subgroups, using subjective and objective biomarkers, including actigraphy.

**Results:**

The MDE-NAP group had more frequently hypersomnia (30.6% vs 8.2% *p* < .001), daytime sleepiness (73.1% vs 41.8%, *p* < .001), daytime fatigue (97.1% vs 88.5%, *p* = .042), family history of depression (68.2% vs 52.2%, *p* = .044), weight gain (42.2% vs 21.6%, *p* = .006), and feelings of guilt (75.0% vs 57.8%, *p* = .027). The unipolar napping group had greater weight gain (48.8% vs 21.4%, *p* = .004) and more hypersomnia (26.1% vs 3.4%, *p* < .001). The bipolar napping group were more frequently women (76.9% vs 52%, *p* = .046), with more suicidal ideation (65.4% vs 37.8%, *p* = .031), sad mood (73.1% vs 43.1%, *p* = .019), hypersomnia (38.5% vs 15.8%, *p* = .04), and guilt (61.9 vs 35.1, *p* = .049). Regarding sleep scales, MDE-NAP had more hypersomnolence symptoms (HSI total score, *p* < .001), with higher frequencies of sleep inertia, daytime sleep, sleepiness, daytime dysfunction, and sleep attacks; and showed longer sleep duration (PSQI sleep duration, *p* = .025), and higher sleep efficiency (PSQI sleep efficiency, *p* = .015). Actigraphy did not reveal significant between-group differences in the subsample with usable recordings (*N* = 90).

**Conclusions:**

Daytime napping during MDE appears to identify a distinct hypersomnolence phenotype with sleep-vegetative burden, supporting the systematic assessment of napping to guide personalized sleep- and circadian-based therapeutic strategies.

*This paper is part of the Sleep and Circadian Mechanisms in Mental Health Collection*.

## Graphical Abstract

**Graphical Abstract:**
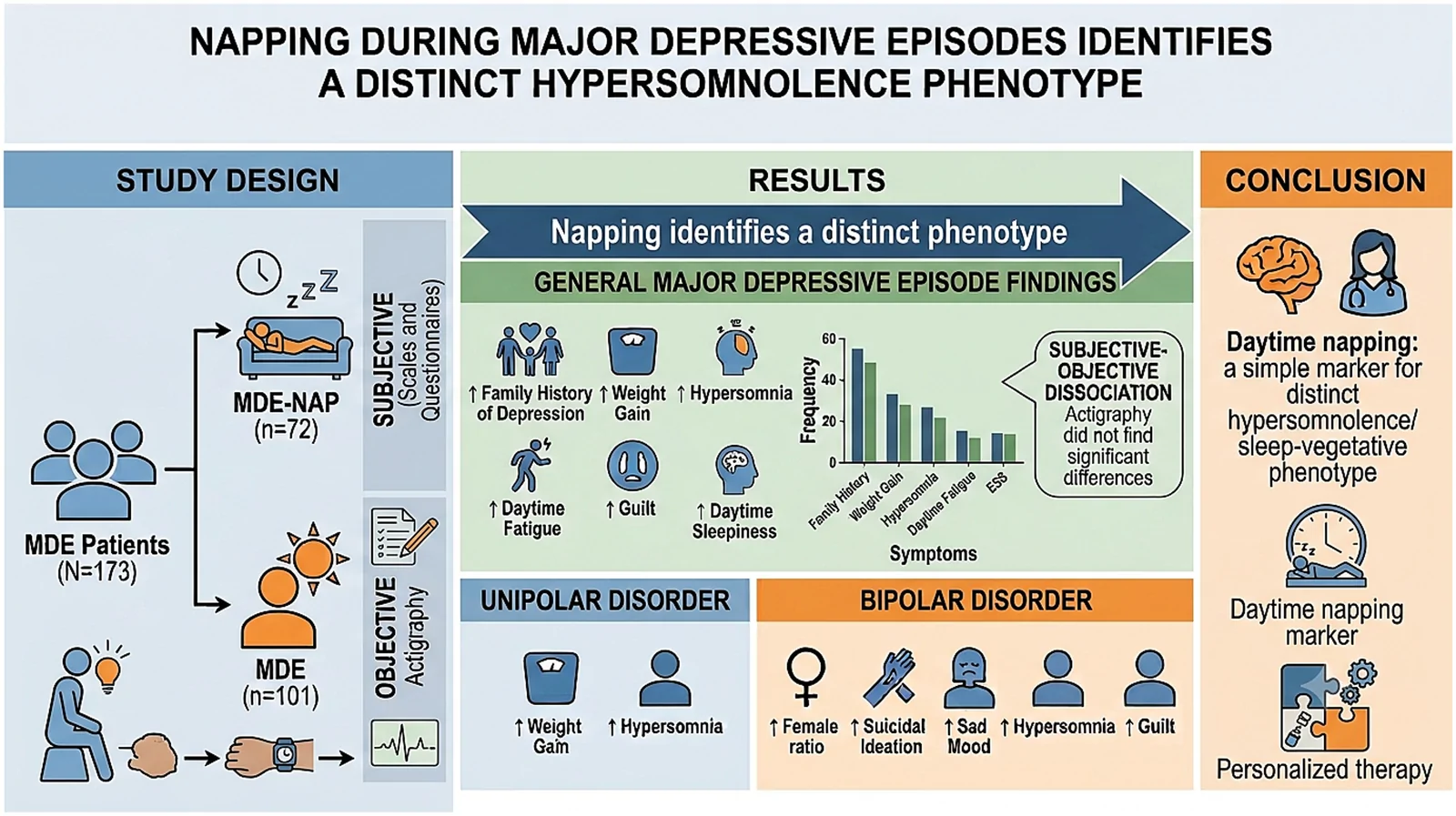

**Statement of Significance**

Daytime napping is common during major depressive episodes, yet its clinical significance remains poorly understood. This study shows that self-reported napping identifies a distinct hypersomnolence and sleep–vegetative phenotype, characterized by greater hypersomnia, daytime sleepiness, fatigue, sleep inertia, daytime dysfunction, and sleep attacks, despite similar overall depression severity. This pattern was observed across unipolar and bipolar depression, with diagnosis-specific clinical features. Interestingly, these marked subjective differences were not accompanied by significant actigraphic differences, highlighting a potential dissociation between experienced hypersomnolence and objective rest–activity measures.

As a simple clinical marker, asking about daytime napping may help identify patients requiring more detailed assessment of hypersomnolence and sleep–wake disturbances and support more personalized sleep- and circadian-informed management.

## Introduction

Major depressive episodes (MDE) affect up to 20 per cent of the general population and are projected to become the leading cause of disability by 2030 according to the World Health Organization [1]. This makes their diagnosis and management appear as a major public health issue. MDE significantly reduces psychosocial functioning and quality of life, and is associated with a high suicide rate [2, 3]. Currently, their diagnosis relies solely on clinical assessment and diagnostic criteria of international diagnostic classifications such as the DSM-5-TR [4]. Despite international efforts to identify biomarkers of MDE, none has been yet implemented in clinical practice for diagnostic purposes or for predicting disease progression or therapeutic response [5]. This limitation is partly explained by the profound heterogeneity of mood disorders, particularly regarding sleep and circadian rhythm disturbances. Over 90 per cent of patients with MDE report sleep disturbances during acute phases [6]. Additionally, current research suggests that these sleep disturbances can occur at any stage of the disorder [7] and are associated with a more unfavorable disease course, increased symptom severity, a higher risk of relapses and recurrences, poorer treatment response, greater therapeutic resistance, and increased suicide risk [3, 8, 9]. Recent large-scale data further highlight the centrality of sleep disturbances in mood disorders trajectories. In a cohort of 13 913 psychiatric inpatients, 81 per cent presented chronic sleep disorders, which were associated with longer and more frequent hospitalizations, as well as increased use of seclusion and physical restraint [10]. Patients with chronic sleep disturbances also displayed higher rates of psychiatric and other medical comorbidities, underscoring sleep as a core marker of illness severity and unfavorable progression [10]. Given this strong link between sleep–wake regulation and mood disorders, understanding specific sleep-related behaviors, such as napping during MDE, is essential. Whether napping represents a compensatory behavior, a marker of disorder severity, or an early sign of altered sleep–wake homeostasis remains unclear, and warrants focused investigation.

While napping is a very common condition for patients with MDE who suffer from excessive daytime sleepiness and fatigue, few studies explored its effects. Some research has reported more severe manifestations of depression in patients who nap [11], while another found that a 30-minute nap reduced anxiety, fatigue and depressive symptoms [12]. Interestingly, it is well recognized that depressive episodes are associated with sleep–wake disturbances and circadian rhythm alterations [13]. Excessive daytime sleepiness and fatigue are also core symptoms of depression and napping may be linked to these and may affect night-time sleep [14]. Caution is required regarding increased time spent in bed, as research indicates that prolonged napping is associated with a higher risk of obesity and chronic diseases, particularly when naps are of longer duration [15].

Moreover, research showed distinct differences in sleep patterns and lifestyle habits between patients with unipolar disorder and those with bipolar disorder. Patients with bipolar disorders in remission, have longer sleep latency, a longer sleep duration and nocturnal awakenings, and lower sleep efficiency [16]. Patients with bipolar disorder often experience hypersomnia during depressive episodes, while unipolar patients are more prone to insomnia and early morning awakenings [17–21]. These findings highlighted the variability in sleep disruptions among bipolar disorder and unipolar disorder. In this context, our analysis focused on patients suffering from unipolar disorder or bipolar disorder separately. We aimed to research how daytime sleep impacts their overall mood regulation, as they may use napping to compensate for poor night-time sleep.

In this context, our main hypothesis is that daytime napping observed during MDE would be associated with more pronounced sleep and circadian disruptions, as well as a distinct clinical profile. We additionally explored whether these associations differed between unipolar and bipolar depression. The aim was to determine whether a simple clinical marker (reported daytime napping) could help characterize heterogeneity in sleep–wake manifestations during MDE.

To our knowledge, this is the first study combining subjective sleep questionnaires, circadian assessments and actigraphy to characterize the clinical significance of daytime napping during MDE.

## Materials and methods

### Study design

This is a prospective case–control study conducted in routine care that first compares patients with MDE across both unipolar and bipolar disorders, distinguishing between those who nap and those who do not. The study then further compares MDE patients who nap within the unipolar and bipolar groups separately. Sleep and circadian rhythm markers are evaluated using both subjective measures (self-report questionnaires) and objective measures (actigraphy for at least 14 days).

Patients were recruited from a routine care cohort from the Psychiatry, Sleep and Chronobiology Reference Center (ChronoS Center), or during hospitalization for MDE at Department of Psychiatry and Addictology, Bichat Claude Bernard Hospital in Paris. Patient were drawn from January 2022 to March 2024. Patients’ medical histories were collected (treatments, recurrences, progression) and, with questionnaires and current MDE characteristics, comorbidities. Data were collected during routine MDE assessments following DSM-5-TR criteria. Patients received an information note explaining that their clinical data could be used for research purposes, with the possibility to decline (non-opposition procedure). This clinicalresearch project entitled “Étude des biomarqueurs du sommeil et des rythmes circadiens dans les troubles psychiatriques « Som-Psy »“ was approved (N°CER-2020–56) by the Ethical Review Committee for Biomedical Research Projects “Comité d’Evaluation de l’Ethique des projets de Recherche Biomédicale (CEERB) Paris Nord” (Institutional Review Board–IRB 00006477–of HUPNVS, Paris 7 University, AP-HP).

### Sociodemographic and clinical parameters

Patients were all adults (18–70 years old) with a diagnosis of MDE (unipolar and bipolar disorders) confirmed by the Structured Clinical Interview for DSM-5-TR [4(p5)]. Napping status was based on patients’ self-report of daytime sleep episodes during the current MDE. Because data were collected in routine care, no prespecified minimum duration, frequency, or timing threshold was used to define napping. Actigraphy was used as a complementary objective assessment of rest–activity patterns but was not used to determine group allocation.

Socio-demographics characteristics such as gender, age, and body mass index (BMI) were collected. Other clinical parameters were assessed including especially the type of mood disorder (unipolar or bipolar), the age of onset, the family history of suicide, suicide attempt (SA) and depression, the personal lifetime history of suicide attempts, the number of hospitalizations, the numbers of MDE, and the severity of depressive symptoms (using the Montgomery-Asberg Depression Rating Scale [MADRS]) [22]. We also recorded current symptoms of depression, which were assessed by psychiatrists during an interview, such as, feelings of guilt, weight loss, weight gain, fatigue, psychomotor retardation, difficulty concentrating or hypersomnia according to the DSM-5-TR criteria.

Finally, we investigated psychiatric, addictive and sleep comorbidities during the psychiatrist interview such as obstructive sleep apnea diagnosis, complaint of hypersomnia (defined by an increased 24-hour sleep duration compared to their usual sleep duration), naps, hyperarousal and inertia defined according to the DSM-5-TR or the ICSD-3 criteria [23, 24]. Psychotropic, hypnotic and antidepressant treatments were also documented. Patients deprived of liberty by administrative or judicial decision were not considered.

### Sleep and circadian rhythms measures

#### Self-report questionnaires

Patients completed the French versions of the following sleep and circadian rhythms questionnaires: insomnia symptoms in the past month were assessed with the Insomnia Severity Index (ISI) [25] with ISI 1A representing the difficulty falling asleep, ISI 1B representing the difficulty maintaining sleep, ISI 1C representing early morning awakening, ISI 2 representing the dissatisfaction with sleep pattern, ISI 3 representing the interference with daily functioning, ISI 4 representing how noticeable the sleep problems are to others and ISI 5 representing the distress caused by sleep issues. Subjective sleep quality was assessed with the Pittsburgh Sleep Quality Index (PSQI) [26], hypersomnia symptoms in the past month were assessed with the Hypersomnia Severity Index (HSI) [27]. The circadian typology was evaluated by the 19-item Morningness–Eveningness Questionnaire (MEQ or Horne & Ostberg score) consisting of 19 questions about preferred sleep time and daily performance. The total score ranges from 16 to 86 and higher score indicates greater morningness chronotype [28]. The Global Seasonality Score (GSS) of the Seasonal Pattern Assessment Questionnaire (SPAQ) [29] was used to assess seasonality, more precisely the seasonal change across 6 dimensions, namely sleep length, social activity, mood, weight, appetite and energy level. The degree of change of the 6 dimensions were assessed by a single item each scored from 0 (no change) to 4 (extremely marked change). The 6 scores were summed up to calculate the general seasonality score (GSS), which thus ranges from 0 (no seasonality) to 24 (extreme seasonality).

Additionally, other questionnaires were used to assess sleep symptoms and characterize patients: The Epworth Sleepiness Scale (ESS) is an instantaneous subjective measure of excessive daytime sleepiness [30]. A total score of ≥11 on the ESS indicates excessive daytime sleepiness.

#### Actigraphy

Patients wore an actigraph watch (MotionWatch 8), on the non-dominant hand for at least 14 consecutives days [31, 32]. This actigraph detects movements over time. Patients were supposed to press when they went to bed and again when they got out of bed to start the day. Simultaneously, they filled out a sleep diary that helped to correct any incongruences between both. The MotionWare 8 software (version 1.3.17) was used to collect and analyze data. In this study we have focused on (1) total night sleep time (TST) measured in minutes, (2) the actigraphic markers of phase with the L5 onset which is the onset of the 5 hours the least active; and the M10 onset which is the onset of the 10 hours the most active, the L5 average represents the activity amplitude during the 5 least actives hours, indicating how restful the sleep periods are, while the M10 average represents activity during the 10 most active hours, indicating the peak of the circadian rhythm, (3) the actual wake time onset (WASO) representing the time spent awake during the sleep period, measured in hours, (4) the sleep efficiency (SE) that represented the total sleep time / time in bed (expressed as a percentage), (5) the sleep onset latency (SOL) which is the time taken to fall asleep after going to bed measured in minutes, (6) the fragmentation index (FI) that represents the degree of fragmentation of the sleep period, (7) the interdaily stability (IS) that represents the degrees of regularity of the activity-rest pattern, and finally (8) the intradaily variability (IV) that represents the degrees of fragmentation in rest-activity periods.

Actigraphy analyses were conducted as complete-case analyses restricted to participants with usable recordings (*N* = 90). Actigraphy variables were not imputed. Missingness in actigraphy therefore reflects availability of usable recordings rather than item-level missing data.

#### Statistical analysis

The datawere coded, validated, and analyzed using R (version 4.2.2) and RStudio (2022.07.2 + 576) software. To assess differences between quantitative variables, we used unpaired parametric t-tests, after checking for normality. To analyze differences between qualitative variables, we used the Chi2 test. For all quantitative variables, an equality of variance test (Fisher test) was performed beforehand. When the variances could be considered significantly different, the Welch test was used to compare the means. In the opposite case, the Student test was used. The results are expressed as the mean and standard deviation, followed by the median and interquartile range if the distribution of values does not follow a normal distribution, with a given confidence index (CI) at 95%. The significance threshold was a *p*-value < .05. We applied the Bonferroni correction only to the primary objectives, which were to compare depressed napping patients with depressed non-napping ones. We did not apply the correction within the unipolar and bipolar subgroups, as these analyses were exploratory in nature. Missing data were ignored if they represented less than 10 per cent of the total number of patients. Otherwise, a method for managing missing data with multiple imputations (MICE) had been used.

## Results

### Sample characteristics

A total of 173 patients were drawn from routine care cohort, including 72 patients who napped (MDE-N) and 101 patients who did not (MDE). We also conducted separate analyses for unipolar and bipolar disorders. Among the 107 patients with unipolar disorder, 46 napped (UD-N) and 61 did not (UD). Among the 66 patients with bipolar disorder, 26 napped (BD-N) and 40 did not (BD). In these tables, significant results (*p* < .05) are in bold.

There were no statistical differences between the group regarding sociodemographic characteristics (Table 1).

**Table 1:** Sociodemographic characteristics in the major depressive episode napping group (MDE-N) compared to the major depressive episode non-napping group (MDE)

	Total sample *N* = 173	MDE-N *N* = 72	MDE *N* = 101	*t-*test ou χ ^2^ or Wilcoxon	*p-value*
	Groups mean (SD) or n (%)				
Age (years)	40.3 (14.9)	39.4 (14.4)	41.0 (15.36)	0.628	.531
Gender				1.829	.176
Male	63 (36.4)	22 (30.6)	41 (40.6)		
Female	110 (63.6)	50 (69.4)	60 (59.4)		
Physical activity				2.274	.132
Yes	94 (58.8)	33 (51.6)	61 (63.5)		
No	66 (41.3)	31 (48.4)	35 (36.5)		
Kids				0.023	.878
Without	94 (64.4)	41 (65.1)	53 (63.9)		
With	52 (35.6)	22 (34.9)	30 (36.1)		
Marital status				0.002	.967
Single	99 (57.6)	41 (57.7)	58 (57.4)		
Married	73 (42.4)	30 (42.3)	43 (426)		
Professional status				0.614	.433
In activity	112 (73.7)	50 (76.9)	62 (71.3)		
Without activity	40 (26.3)	15 (23.1)	25 (28.7)		
BMI (kg/m^2^)	26.3 (6.8)	27.6 (6.4)	25.4 (7.0)	1.73	.086
Coffee consumption				0.134	.714
Yes	108 (67.1)	44 (68.8)	64 (66.0)		
No	53 (32.9)	20 (31.3)	33 (34.0)		
Tea consumption				0.092	.762
Yes	47 (29.0)	18 (27.7)	29 (29.9)		
No	115 (71.0)	47 (72.3)	68 (70.1)		
Smoking				0.068	.794
Yes	41 (30.4)	18 (31.6)	23 (29.5)		
No	94 (69.6)	39 (68.4)	55 (70.5)		
Alcohol				0.145	.703
Yes	26 (15.1)	10 (13.9)	16 (16.0)		
No	146 (84.9)	62 (86.1)	84 (84.0)		
Antidepressant treatments				2.543	.111
Yes	106 (62.0)	49 (69.0)	57 (57.0)		
No	65 (38.0)	22 (31.0)	43 (43.0)		
Hypnotical treatments				2.141	.143
Yes	67 (39.4)	23 (32.9)	44 (44.0)		
No	103 (60.6)	47 (67.1)	56 (56.0)		
Anxiolytic treatments				0.008	.927
Yes	76 (44.7)	31 (44.3)	45 (45.0)		
No	94 (55.3)	39 (55.7)	55 (55.0)		
Antipsychotic treatments				0.719	.397
Yes	16 (9.4)	5 (7.1)	11 (11.0)		
No	154 (90.6)	65 (92.9)	89 (89.0)		
Mood stabilizer treatments				0.511	.475
Yes	48 (28.1)	22 (31.0)	26 (26.0)		
No	123 (71.9)	49 (69.0)	74 (74.0)		

MDE = major depressive episode; MDE-N = major depressive episode with nap; BMI = body mass index.

Regarding clinical characteristics (Table 2, Figure 1), patients in the MDE-N group, compared to the MDE group more frequently had a family history of depression (68.2% vs 52.2%). They had a greater weight gain (42.2% vs 21.6%), had more frequently hypersomnia (30.6% vs 8.2%), more daytime fatigue (97.1% vs 88.5%), an increased feeling of guilt (75.0% vs 57.8%), and increased daytime sleepiness (73.1% vs 41.8%).

**Table 2:** Clinical characteristics in the major depressive episode napping group (MDE-N) compared to the major depressive episode non-napping group (MDE)

Clinical characteristics	Total sample *N* = 173	MDE-N *N* = 72	MDE *N* = 101	*t*-test ou χ ^2^ or Wilcoxon	*p-value*
	Groups mean (SD) or n (%)				
Age of onset (years)	26.4 (12.6)	25.6 (11.2)	27.1(13.7)	0.702	.484
Age of first hospitalization	32.1 (16.1)	31.8 (16.9)	32.3 (15.9)	0.127	.899
Number of MDE	3.64 (4.36)	3.77 (5.30)	3.53 (3.49)	0.29	.772
Number of hospitalization	1.65 (3.62)	1.34 (4.25)	1.89 (3.06)	0.57	.57
Suicide attempt				0.048	.827
Yes	42 (24.9)	18 (25.7)	24 (24.2)		
No	127 (751)	52 (74.3)	75 (75.8)		
Suicidal ideation				1.847	.174
Yes	96 (56.5)	45 (62.5)	51 (52.0)		
No	74 (43.5)	27 (37.5)	47 (48.0)		
Family history of depression				4.067	**.044**
Yes	93 (58.9)	45 (68.2)	48 (52.2)		
No	65 (41.1)	21 (31.8)	44 (47.8)		
Family history of suicide				2.873	.09
Yes	31 (19.1)	17 (25.4)	14 (14.7)		
No	131 (80.9)	50 (74.6)	81 (85.3)		
Sad mood				1.077	.299
Yes	120 (71.9)	54 (76.1)	66 (68.8)		
No	47 (28.1)	17 (23.9)	30 (31.3)		
Loss of interest				1.828	.176
Yes	125 (76.2)	57 (81.4)	68 (72.3)		
No	39 (23..8)	13 (18.6)	26 (27.7)		
Weight loss				0.014	.907
Yes	40 (26.5)	17 (27.0)	23 (26.1)		
No	111 (73.5)	46 (73.0)	65 (73.9)		
Weight gain				7.448	**.006**
Yes	46 (30.3)	27 (42.2)	19 (21.6)		
No	106 (69.7)	37 (57.8)	69 (78.4)		
Hypersomnia				14.085	**< .001**
Yes	30 (17.8)	22 (30.6)	8 (8.2)		
No	139 (82.2)	50 (69.4)	89 (91.8)		
Insomnia				2.724	.099
Yes	146 (86.9)	59 (81.9)	87 (90.6)		
No	22 (13.1)	13 (18.1)	9 (9.4)		
Psychomotor retardation				0.816	.366
Yes	80 (51.9)	31 (47.7)	49 (55.1)		
No	74 (48.1)	34 (52.3)	40 (44.9)		
Fatigue				4.149	**.042**
Yes	153 (92.2)	68 (97.1)	85 (88.5)		
No	13 (7.8)	2 (2.9)	11 (11.5)		
Feelings of guilt				4.872	**.027**
Yes	100 (64.9)	48 (75.0)	52 (57.8)		
No	54 (35.1)	16 (25.0)	38 (42.2)		
Difficulty concentrating				0.102	.749
Yes	129 (80.6)	54 (81.8)	75 (79.8)		
No	31 (19.4)	12 (18.2)	19 (20.2)		
Seasonality				0.941	.332
Yes	37 (21.6)	13 (18.1)	24 (24.2)		
No	134 (78.4)	59 (81.9)	75 (75.8)		
Subtype				0.38	.538
Unipolar	107 (73.3)	46 (70.8)	61 (75.3)		
Bipolar	39 (26.7)	19 (29.2)	20 (24.7)		
Panic disorder				3.408	.065
Yes	49 (28.3)	15 (20.8)	34 (33.7)		
No	124 (71.7)	57 (79.2)	67 (66.3)		
PTSD				0.648	.421
Yes	75 (43.9)	29 (40.3)	46 (46.5)		
No	96 (56.1)	43 (59.7)	53 (53.5)		
Daytime sleepiness				15.721	**< .001**
Yes	90 (54.5)	49 (73.1)	41 (41.8)		
No	75 (45.5)	18 (26.9)	57 (58.2)		
Obstructive sleep apnea syndrome				0.179	.672
Yes	52 (34.0)	24 (35.8)	28 (32.6)		
No	101 (66.0)	43 (64.2)	58 (67.4)		
Parasomnia				0.006	.941
Yes	68 (39.8)	28 (39.4)	40 (40.0)		
No	103 (50.2)	43 (60.6)	60 (60.0)		

MDE = major depressive episode; MDE-N = major depressive episode with nap; BMI = body mass index; PTSD = post traumatic stress disorder.

**Figure 1:**
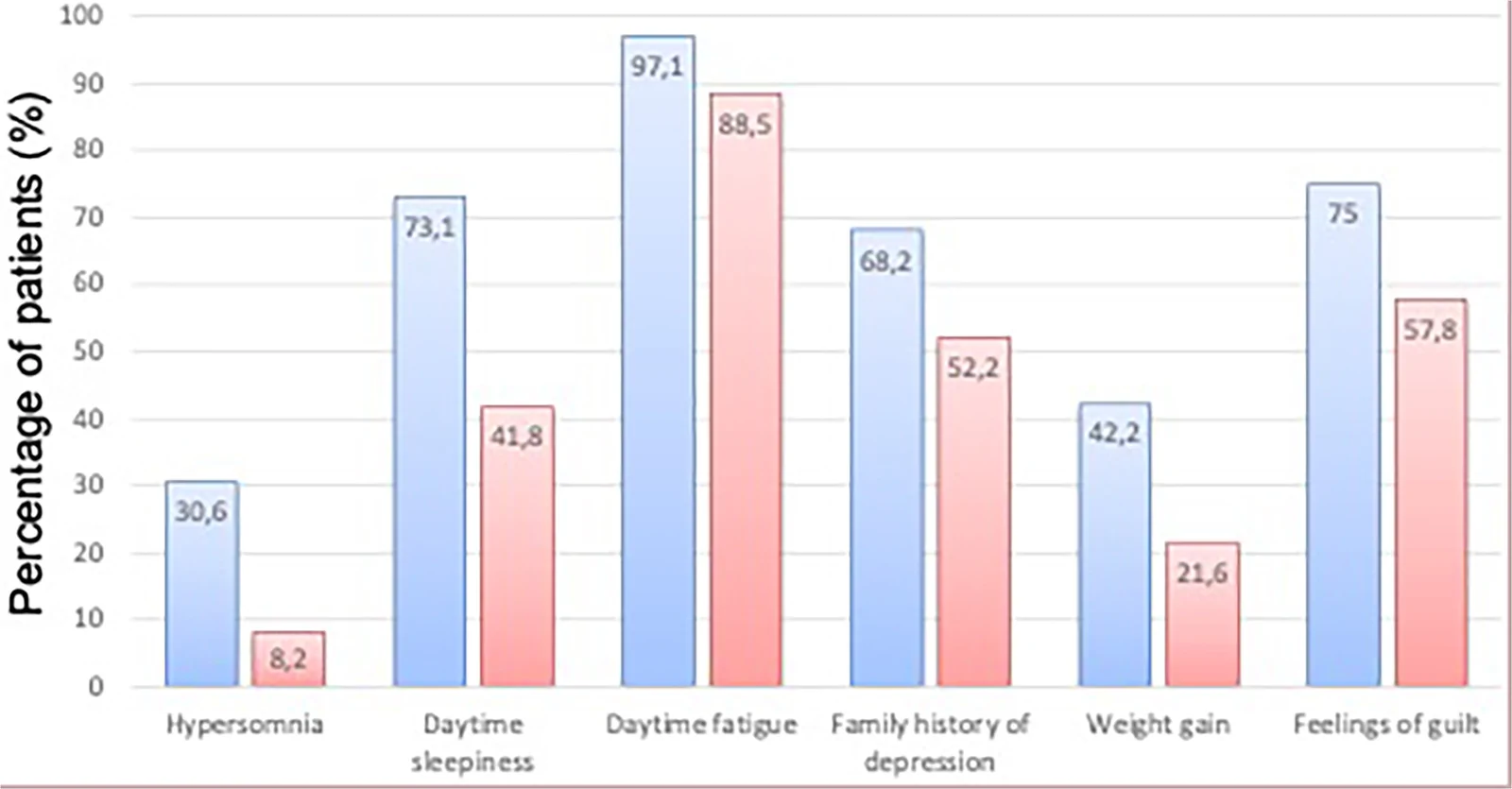
Clinical Characteristics in Depressed Patients With and Without Napping.

In the subgroup analysis that included only patients with unipolar disorders (UP), the napping group (UP-N), compared to the non-napping unipolar group (UP), had greater weight gain (48.8% vs 21.4%), more frequently hypersomnia, as well as daytime sleepiness, and daytime fatigue (Table S1).

In the subgroup analysis focusing solely on patients with bipolar disorder (BD), the napping group (BD-N), compared to the non-napping bipolar group (BD), show higher proportion of women, increased suicidal ideation, sadness, hypersomnia, daytime sleepiness, daytime fatigue, and feelings of guilt (Table S2).

### Sleep and circadian rhythms assessment

Regarding sleep and circadian rhythm subjective analyses (Table 3), the MDE-N group, compared to the MDE group, showed statistically significant differences with more frequent hypersomnia symptoms (HSI total score, *p* < .001) (Table 3). Among hypersomnia symptoms, patients from the napping group reported more frequently sleep inertia (HSI 1b, *p* < .015), sleep during day (naps) (HSI 1c, *p* < .001), sleepiness (HSI 1d, *p* = .026), “daytime dysfunction”, defined as problems that interfere with the daily functioning, for instance daytime fatigue, ability to function at work or in daily chores, concentration (HSI 3, *p* = .013), and “sleep attacks”, defined as unintended sleep on inappropriate situations (HIS 6, *p* = .018). Regarding sleep quality, the napping group, in comparison with the non-napping group, showed statistically significant differences with longer sleep duration (PSQI sleep duration, *p* = .025), and higher sleep efficiency (PSQI sleep efficiency, *p* = .015).

**Table 3:** Comparison regarding subjective sleep and circadian rhythm markers assessed with self-report questionnaires in the major depressive episode napping group (MDE-N) and the major depressive episode non-napping group (MDE)

	Total sample *n* = 173	MDE-N *n* = 72	MDE *n* = 101	*t*-test or Wilcoxon	*p-value*
	Groups mean (SD) or n (%)				
Depressive symptoms (MADRS)	24.37 (8.32)	23.53 (8.58)	25.02 (8.12)	0.931	.354
Sleep disturbances (PSQI total score)	12.38 (4.17)	11.54 (3.80)	12.97 (4.35)	1.915	.058
PSQI-sleep subjective quality	2.23 (0.75)	2.18 (0.81)	2.26 (0.72)	0.511	.61
PSQI-sleep latency	1.95 (1.04)	1.80 (1.04)	2.06 (1.03)	1.351	.18
PSQI-sleep duration	1.06 (1.18)	0.78 (1.09)	1.26 (1.21)	2.27	**.025**
PSQI-sleep efficiency	1.39 (1.22)	1.06 (1.20)	1.61 (1.20)	2.482	**.015**
PSQI-sleep disturbance	1.78 (0.63)	1.82 (0.63)	1.76 (0.62)	0.504	.615
PSQI-sleep promoting medication	2.10 (1.26)	1.96 (1.35)	2.20 (1.20)	1	.32
PSQI-sleep dysfunction	1.82 (0.76)	1.82 (0.86)	1.83 (0.68)	0.083	.934
Seasonality (GSS)	10.07 (6.27)	11.67 (6.07)	8.89 (6.20)	2.526	**.013**
Epworth Spleepiness Scale (ESS)	9.06 (5.36)	11.73 (5.09)	7.05 (4.67)	5.327	**< .001**
Insomnia symptoms (ISI total score)	16.82 (7.43)	16.43 (7.05)	17.14 (7.75)	0.564	.574
ISI 1A (Difficulty Falling Asleep)	2.37 (1.26)	2.00 (1.22)	2.68 (1.21)	3.077	**.003**
ISI 1B (Difficulty Maintaining Sleep)	2.35 (1.26)	2.14 (1.35)	2.51 (1.15)	1.626	.107
ISI 1C (Early Morning Awakening)	2.45 (1.36)	2.46 (1.42)	2.45 (1.33)	0.003	.997
ISI 2 (Dissatisfaction with sleep pattern)	3.24 (0.89)	3.23 (0.81)	3.25 (0.96)	0.136	.892
ISI 3 (Interference with daily functioning)	3.15 (0.83)	3.21 (0.68)	3.10 (0.94)	0.777	.439
ISI 4 (Noticeability due to sleep issues)	2.46 (1.06)	2.38 (0.87)	2.52 (1.20)	0.752	.454
ISI 5 (Distress caused by sleep issues)	2.83 (0.96)	2.80 (0.94)	2.86 (0.98)	0.327	.744
Chronotype (Horne et Otsberg questionnaire, MEQ)	45.75 (11.40)	45.13 (11.11)	46.23 (11.69)	0.522	.603
Hypersomnia Severity Index total	17.90 (6.15)	20.08 (5.01)	15.87 (6.35)	3.611	**< .001**
HSI 1A–Sleep duration	0.99 (1.35)	1.23 (1.53)	0.78 (1.15)	1.498	.139
HSI 1B–Sleep inertia	2.31 (1.31)	2.68 (1.116)	2.00 (1.37)	2.488	**.015**
HSI 1C–Sleep during day	1.41 (1.39)	2.10 (1.30)	0.83 (1.20)	4.705	**< .001**
HSI 1D–Sleepiness	1.92 (1.21)	2.23 (0.97)	1.66 (1.34)	2.273	**.026**
HSI 2–Satisfaction	2.70 (1.38)	2.83 (1.26)	2.60 (1.48)	0.78	.438
HSI 3–Daytime dysfunction	3.06 (0.97)	3.33 (0.62)	2.83 (1.15)	2.557	**.013**
HSI 4–Symptoms apparentness	2.35 (1.16)	2.45 (1.01)	2.26 (1.27)	0.767	.445
HSI 5–Distress	2.77 (0.97)	2.78 (0.89)	2.77 (1.05)	0.044	.965

MADRS = Montgomery-Asberg Depression Rating Scale; PSQI = Pittsburgh Sleep Quality Index. GSS = General Seasonality Score; ESS = Epworth Sleepiness Scale; ISI = Insomnia Severity Index; HSI = Hypersomnia Severity Index.

Regarding seasonal effects, the napping group showed a significantly higher GSS score on the SPAQ than the non-napping group, indicating more pronounced seasonal variations in sleep, social activity, mood, weight, appetite, and energy. No difference was observed in chronotype (MEQ).

Regarding analyses in patients with unipolar depression (Table S3), the napping group, in comparison with the non-napping group, showed statistically significant differences with more frequent hypersomnia symptoms (HSI, *p* < .001). Among hypersomnia symptoms, patient in the napping group reported more frequently sleep inertia (HIS 1b, *p* = .025), sleep during day (HSI 1c, *p* < .001), sleepiness (HIS 1d, *p* = .058), daytime dysfunction (HSI 3, *p* = .067), and sleep attacks (HSI 6, *p* = .014). Regarding sleep quality, the napping group, in comparison with the non-napping group, showed statistically significant differences with longer sleep duration (PSQI sleep duration, *p* = .049), and higher sleep efficiency (PSQI sleep efficiency, *p* = .023). The Epworth Severity Scale (ESS), in the napping group, in comparison with the non-napping group, showed statistically significant differences with more daytime sleepiness (*p* < .01). The Insomnia Severity Index (ISI), in the non-napping group, complained more about difficulty falling asleep than the napping group (ISI 1A, *p* = .002). No difference was observed in chronotype (MEQ).

Regarding analyses in patients with bipolar depression (Table S4), the napping group, in comparison with the non-napping group, showed more hypersomnia symptoms, with more frequent sleep during day (naps) (HSI 1c, *p* = .037), and daytime dysfunction (HSI 3, *p* = .023). Regarding sleep quality, the napping group, in comparison with the non-napping group, showed statistically significant differences with shorter sleep latency (PSQI sleep latency, *p* = .008). The Insomnia Severity Index (ISI), in the non-napping group, complained more about difficulty falling asleep than the napping group (ISI 1A, *p* = .004). No difference was observed regarding the chronotype (MEQ).

### Actigraphy

Actigraphy analyses were performed as complete-case analyses in the 90 participants with usable recordings. No significant differences were observed between the groups (Table 4).

**Table 4:** Comparison of the major depressive episode napping group (MDE-N) with the major depressive episode non napping group (MDE) regarding actigraphy data

	Total sample *N* = 90	MDE-N *N* = 43	MDE *N* = 47	*t*-test or Wilcoxon	*p-value*
	Groups mean (SD) or n (%)				
Total sleep time (TST) (hh:mm:ss)	7:11:16 (1:23:35)	7:08:06 (1:15:29)	7:18:40 (1:30:25)	0.604	.548
Wake after sleep onset (WASO) hh:mm:ss	1: 23: 30 (0: 33: 50)	1: 30: 13 (0: 30: 34)	1: 17: 14 (0: 35: 50)	1.863	.066
Sleep onset latency (SOL)	80.85 (7.28)	79.61 (6.42)	82.01 (7.90)	1.591	.115
Sleep efficiency (SE) %	80.94 (7.26)	79.83 (6.51)	81.99 (7.82)	1.449	.151
Fragmentation Index FI	32.51 (13.05)	34.86 (13.43)	30.30 (12.41)	1.699	.093
Interdaily stability (IS)	0.38 (0.12)	0.37 (0.11)	0.40 (12)	1.196	.235
Intradaily variability (IV)	0.88 (0.20)	0.88 (0.24)	0.89 (0.17)	0.221	.826
L5 Start Hour (hh: mm: ss)	3:36:00 (5:49:01)	4:00:00 (6:16:45)	3:14:03 (5:24:28)	0.623	.538
L5 average	1091 (929)	1055 (661)	1125 (1130)	0.367	.715
M10 Start Hour (hh: mm:ss)	10:22:13 (02:07:36)	10:22:20 (2:17:58)	10:22:59 (1:47:18)	< 0.001	.98
M10 average	11 849 (4976)	11 859 (4634)	11 839 (5326)	0.019	.985

TST = Total Sleep Time; WASO = Wake After Sleep Onset; SOL = Sleep Onset Latency; SE = Sleep efficiency; FI = Fragmentation Index; IS = Interdaily Stability; IV = Intradaily Variability.

## Discussion

In this study, self-reported daytime napping during a MDE was consistently associated with a distinct hypersomnolence phenotype across diagnoses. Napping appears as an easily identifiable clinical marker associated with a broader hypersomnolence profile. Indeed, patients who napped reported more hypersomnia symptoms, greater daytime sleepiness and fatigue, and broader alterations of daytime alertness, suggesting that napping may reflect or contribute to poorer nocturnal sleep quality, characterized by longer duration and increased efficiency. They also presented more frequent family history of depression, alongside greater weight gain and increased guilt. When examining diagnostic subgroups, distinct patterns emerged. Among individuals with unipolar depression, napping was specifically associated with greater weight gain and more pronounced hypersomnia. In contrast, in the bipolar depression subgroup, nappers were more often women and exhibited higher levels of suicidal ideation than non-nappers. Patients with bipolar depression who napped also showed more hypersomnia symptoms, shorter sleep latency, reinforcing the hypothesis of a deeper dysregulation of sleep–wake homeostasis. Overall, the convergence of hypersomnia, daytime alertness complaints, and altered nocturnal sleep parameters across both unipolar and bipolar depression suggests that napping may represent a transdiagnostic symptom of a distinct hypersomnolence and sleep-vegetative phenotype, relevant for clinical assessment and treatment planning. Finally, interestingly, no significant between-group differences were observed on actigraphy-derived measures, suggesting that the napping marker might be primarily reflected by subjective hypersomnolence and daytime impairment rather than objective sleep–wake parameters. Given the smaller actigraphy sample (*N* = 90), these null findings should be interpreted cautiously.

These findings align with previous research that has highlighted the high prevalence of sleep disturbance prior to a mood episode in patients with bipolar disorder with differences between depressive and manic episodes [33]. This underlines the importance of looking at sleep quality in patients with mood disorders to detect relapses as early as possible [8]. The literature has proved that MDE is reported with sleep complaints, combining night-time and daytime disturbances, with co-occurrence of insomnia (especially early wake-up) and hypersomnia, this latter associated with a two-/three-fold increased risk of bipolar disorders [6]. This is consistent with the need to pay close attention to sleep complaints during psychiatric interviews. The longitudinal follow-up of these patients will enable a better understanding of the evolution of such residual symptoms. On the other hand, patients with bipolar disorders have residual symptoms, chronic perturbations that have a major impact on levels of functioning, like sleep disturbances [34]. The combination of hypersomnia and weight gain overlaps with the DSM-5-TR atypical-features specifier. However, atypical-features status was not systematically recorded, and key defining features such as mood reactivity and rejection sensitivity were not consistently available. The napping-associated profile therefore cannot be considered equivalent to atypical depression. Rather, napping may identify a partially overlapping sleep-vegetative dimension that warrants prospective characterization using formal atypical-features criteria. Finally, there are many studies looking at the repercussions of depression on sleep, but very few studying the impact of napping. Actually, a study found a correlation between daytime napping and increased depressive symptoms in older Chinese adults [35], while another one revealed that the timing of naps played a crucial role in their impact on depressive symptomatology, with morning naps being particularly detrimental [36]. We know that sleep stages vary according to the length of night, but also according to the presence of a MDE. The onset latency of REM sleep is shorter [37, 38]. REM sleep alterations observed in depression are considered biological markers of the condition, and previous studies have shown that REM sleep deprivation can reduce depressive symptoms [39, 40]. This suggests that REM sleep may hinder the regulation of emotional processing in depressed individuals, potentially contributing to the neurocognitive distortions observed in this disorder [41, 42]. Another study examined the effect of napping on facial perception and emotion representation in depressed individuals compared to non-depressed controls. Depressed patients who took a 90-minute nap (with REM sleep present) perceived emotions more intensely, with faces being seen as angrier [43].

Our findings are particularly significant because we found that depressed patients who take naps are not more likely to suffer from insomnia, but instead exhibit more symptoms of hypersomnolence dimensions [44]. Hypersomnolence, which encompasses hypersomnia, sleep inertia, excessive daytime sleepiness, and napping, should be thoroughly assessed in depressed patients, as it is associated with a hypersomnolence and sleep-vegetative phenotype. Previous research has shown that hypersomnolent patients tend to have a higher recurrence of the disease, increased suicidal ideation, and a greater number of medical comorbidities. Additionally, hypersomnia has been identified as a predictive factor for depression [45]. Also, a study observed that depressed patients suffering from insomnia and excessive daytime sleepiness were associated with more severe clinical profiles than depressed patients suffering from insomnia without excessive daytime sleepiness [9]. These studies all converge on the idea that hypersomnolence represents a particular subgroup of patients. Another study showed that excessive sleepiness is not associated with longer sleep or bedrest but predicts relapse to mania/hypomania in bipolar disorders [46]. Clinically, assessment of daytime napping may prompt a more detailed evaluation of hypersomnolence, sedating medications, comorbid sleep disorders, time in bed, and sleep–wake regularity. Such assessment may help individualize sleep and circadian-based management, although interventional studies are required before specific treatment recommendations can be derived from these findings.

In contrast with the marked differences observed in subjective sleep complaints and daytime functioning, actigraphy did not reveal significant between-group differences in objective sleep or circadian parameters. This subjective–objective dissociation should be interpreted cautiously. Actigraphy, while well suited to assess global rest–activity rhythms [47] and sleep duration under naturalistic conditions [32, 48], may lack sensitivity to capture qualitative aspects of hypersomnolence, such as sleep inertia, daytime dysfunction, or sleep attacks, which emerged as core features of the napping group. Moreover, daytime napping may partially compensate for nocturnal sleep disturbances, thereby attenuating objective differences in night-time sleep parameters. Finally, usable actigraphy was available in only 90 participants, limiting power to detect modest between-group effects. Thus, the absence of significant actigraphic differences may reflect limited sensitivity to these dimensions and/or insufficient statistical power rather than evidence of an absence of objective sleep–wake differences.

However, our study has several limitations. First, the cross-sectional observational design precludes causal or directional inference: napping may precede, accompany, or result from hypersomnolence and other depressive symptoms. Second, napping status was self-reported and was not defined using prespecified thresholds for duration, frequency, or timing, limiting reproducibility and preventing detailed characterization of nap behavior. Third, residual confounding cannot be excluded. Age, BMI, psychotropic treatments with sedating or activating properties, OSA and other sleep disorders may influence both napping and sleep-related outcomes. Although global MADRS severity did not differ between groups, symptom-level differences may also reflect dimensions of depression not fully captured by the total score. Fourth the small number of patients in the bipolar group represents a limitation, reducing the power of the study, reflecting the lower prevalence of bipolar disorders in the general population [49](42). Another limitation is the multiplicity of tests due to the increased alpha risk. However, we have performed a Bonferroni correction on the results of the main analysis of the MDE patients’ group. Additionally, the difference in treatment between the two groups, with unipolar depression patients primarily treated with antidepressants and bipolar patients with mood stabilizers, could influence the results due to the sedative or stimulating effects of certain medications on sleep architecture [50, 51]. Depending on the antidepressants used, there can be a sedative effect of the molecule (such as Mirtazapine) or a stimulating effect (such as Serotonin and Norepinephrine Reuptake Inhibitors). These antidepressant treatments also modify the sleep architecture with a decrease in REM sleep. Furthermore, we use actigraphy and no polysomnography (PSG), which remains the gold standard assessment. Actigraphy does not allow examination of sleep architecture and may under or over report some aspects of the sleep–wake cycle. Using a PSG could give us further information, like the presence of sleep apnea syndrome. Nevertheless, in our study, and with patients with psychiatric disorders, actigraphy allows recordings across naturalistic conditions. It can record data over a longer duration that PSG, is less expensive and minimally disruptive. A study showed that the comparison between actigraphy and PSG showed quite a good contingency coefficient value (C = 0.64), for patients with insomnia symptoms [52]. These limitations are balanced by the multimodal characterization of sleep and circadian function in a naturalistic clinical cohort and by the use of validated questionnaires together with prolonged actigraphy.

## Conclusion

In conclusion, self-reported daytime napping during MDE was associated with a distinct hypersomnolence and sleep–vegetative phenotype, despite no difference in global MADRS severity. Napping may therefore represent a simple clinical marker prompting more detailed assessment of hypersomnolence and sleep–wake disturbances. Prospective studies with standardized nap characterization and multivariable analyses are needed to determine the independence, temporal direction, and therapeutic relevance of these associations.

## Supplementary Material

SUPPLEMENTAL_MATERIAL_-_final_preview_zpag097

## Data Availability

The data underlying this article are not publicly available because they contain information that could compromise the privacy of patients. De-identified data will be made available by the corresponding author upon reasonable request, subject to institutional and ethical approval.
