# Supplementary material for: Napping during major depressive episodes identifies a distinct hypersomnolence phenotype
Source: Sleep Adv. 2026 Sep 18;7(4):zpag097. doi: 10.1093/sleepadvances/zpag097 (PMC13660474; doi:10.1093/sleepadvances/zpag097)
Supplement: Supplementary file 1 [file supplemental_material_-_final_preview_zpag097.docx]

**SUPPLEMENTAL MATERIAL**

**Napping during major depressive episodes identifies a distinct Hypersomnolence phenotype**

Pierre Alexis **GEOFFROY**^1,2,3,4^; Célia **HEILIGENSTEIN**^5,6^ **;** Sibylle **MAURIES**^1,3^; Justin **ULM**^7^ ; Fériel **ZEHANI**^1,3^; Chantal **HENRY**^2,8^, Michel **LEJOYEUX**^1,2,3^; Patrice **BOURGIN**^4,5,6^; Julia **MARUANI**^1,2,3^.

1.	Département de psychiatrie et d'addictologie, AP-HP, GHU Paris Nord, DMU Neurosciences, Hôpital Bichat - Claude Bernard, F-75018 Paris, France

2.	Université Paris Cité, NeuroDiderot, Inserm, F-75019 Paris, France

3.	Centre ChronoS, GHU Paris - Psychiatry & Neurosciences, 1 rue Cabanis, 75014 Paris, France

4.	CNRS UPR 3212, Institute for Cellular and Integrative Neurosciences, F–67000, Strasbourg University, France

5. Hôpitaux Universitaires de Strasbourg, Service de Pédopsychiatrie de l’Enfant et de l’Adolescent Strasbourg

6. CIRCSom (International Research Center for ChronoSomnology) & Sleep Disorders Center, Strasbourg University Hospital, 1 place de l’Hôpital, F-67000 Strasbourg, France

7. Emergency Department, Percy military teaching hospital, 101 avenue Henri Barbusse 92140 Clamart, France

8. Service Hospitalo Unievrsitaire (SHU), GHU Paris - Psychiatry & Neurosciences, 1 rue Cabanis, 75014 Paris, France

**Corresponding author**:

Pr. Pierre Alexis GEOFFROY

University Hospital Bichat-Claude Bernard

Department of Psychiatry and Addictive Medicine

46 rue Henri Huchard,

75018, Paris, France.

: +33 (0)1 40 25 82 60

E-mail: [pierre.a.geoffroy@gmail.com](mailto:pierre.a.geoffroy@gmail.com)

ORCID : 0000-0001-9121-209X

Table S1. Demographic and clinical characteristics in the unipolar napping group (UD-N) compared to the unipolar non-napping group (UD).

| Socio-demographic characteristics | Total sample  N = 107 | UD-N  N = 46 | UD  N = 61 | t-test ou **χ^2^** or Wilcoxon | *p-value* |
| --- | --- | --- | --- | --- | --- |
|  | Groups mean (SD) or n (%) | | |  |  |
| Age (years) | 39.2 (15.3) | 37.7 (13.5) | 40.5 (16.3) | 1.311 | 0.193 |
| Gender |  |  |  | 0.019 | 0.891 |
| Male | 38 (35.5) | 16 (34.8) | 22 (36.1) |  |  |
| Female | 69 (64.5) | 30 (65.2) | 39 (63.9) |  |  |
| Physical activity |  |  |  | 1.842 | 0.175 |
| Yes | 57 (54.3) | 21 (46.7) | 36 (60.0) |  |  |
| No | 48 (45.7) | 24 (53.3) | 24 (40.0) |  |  |
| Kids |  |  |  | 0.011 | 0.917 |
| Without | 54 (62.8) | 23 (62.2) | 31 (63.3) |  |  |
| With | 32 (37.2) | 14 (37.8) | 18 (3607) |  |  |
| Marital status |  |  |  | 0.857 | 0.355 |
| Single | 62 (58.5) | 24 (53.3) | 38 (62.3) |  |  |
| Married | 44 (41.5) | 21 (46.7) | 23 (37.7) |  |  |
| Professional status |  |  |  | 1.854 | 0.173 |
| In activity | 69 (64.5) | 33 (71.7) | 36 (59.0) |  |  |
| Without activity | 38 (35.5) | 13 (28.3) | 25 (41.0) |  |  |
| BMI (kg/m2) | 26.6 (7.2) | 28.2 (7.3) | 25.5 (7.5) | 1.928 | 0.057 |
| Coffee consumption |  |  |  | 0.089 | 0.766 |
| Yes | 70 (66.0) | 29 (64.4) | 41 (67.2) |  |  |
| No | 36 (34.0) | 16 (35.6) | 20 (32.8) |  |  |
| Tea consumption |  |  |  | 0.032 | 0.859 |
| Yes | 32 (30.2) | 14 (31.1) | 18 (29.5) |  |  |
| No | 74 (69.8) | 31 (68.9) | 43 (70.5) |  |  |
| Smoking |  |  |  | 0.028 | 0.866 |
| Yes | 23 (28.0) | 11 (28.9) | 12 (27.3) |  |  |
| No | 59 (72.0) | 27 (71.1) | 32 (72.7) |  |  |
| Alcohol |  |  |  | 0.231 | 0.63 |
| Yes | 16 (15.0) | 6 (13.0) | 10 (16.4) |  |  |
| No | 91 (85.0) | 40 (87.0) | 51 (83.6) |  |  |
| Antidepressant treatments |  |  |  | 2.093 | 0.148 |
| Yes | 81 (75.7) | 38 (82.6) | 43 (70.5) |  |  |
| No | 26 (24.3) | 8 (17.4) | 18 (29.5) |  |  |
| Hypnotical treatments |  |  |  | 0.814 | 0.367 |
| Yes | 43 (40.6) | 16 (35.6) | 27 (44.3) |  |  |
| No | 63 (59.4) | 29 (64.4) | 34 (55.7) |  |  |
| Anxiolytic treatments |  |  |  | 0.296 | 0.587 |
| Yes | 48 (45.3) | 19 (42.2) | 29 (47.5) |  |  |
| No | 58 (54.7) | 26 (57.8) | 32 (52.5) |  |  |
| Antipsychotic treatments |  |  |  | 0.335 | 0.563 |
| Yes | 9 (8.5) | 3 (6.7) | 6 (9.8) |  |  |
| No | 97 (91.5) | 42 (93.3) | 55 (90.2) |  |  |
| Mood stabilizer treatments |  |  |  | 0.761 | 0.383 |
| Yes | 15 (14.0) | 8 (17.4) | 7 (11.5) |  |  |
| No | 92 (86.0) | 38 (82.6) | 54 (88.5) |  |  |
| Clinical characteristics | Total sample  N = 107 | UD-N  N = 46 | UD  N = 61 | t-test ou χ2 or Wilcoxon | p-value |
| Panic disorder |  |  |  | 1.816 | 0.178 |
| Yes | 33 (30.8) | 11 (23.9) | 22 (36.1) |  |  |
| No | 74 (69.2) | 35 (76.1) | 39 (63.9) |  |  |
| PTSD |  |  |  | 0.466 | 0.495 |
| Yes | 57 (53.8) | 23 (50.0) | 34 (56.7) |  |  |
| No | 49 (46.2) | 23 (50.0) | 26 (43.3) |  |  |
| Hyperarousal |  |  |  | < 0.001 | 0.997 |
| Yes | 67 (64.4) | 29 (64.4) | 38 (64.4) |  |  |
| No | 37 (35.6) | 16 (35.6) | 21 (35.6) |  |  |
| Sleep inertia |  |  |  | 1.565 | 0.211 |
| Yes | 18 (17.3) | 10 (22.7) | 8 (13.3) |  |  |
| No | 86 (82.7) | 34 (77.3) | 52 (86.7) |  |  |
| Daytime sleepiness |  |  |  | 10.37 | 0.001 |
| Yes | 63 (60.0) | 35 (77.8) | 28 (46.7) |  |  |
| No | 42 (40.0) | 10 (22.2) | 32 (53.3) |  |  |
| Obstructive sleep apnea syndrome |  |  |  | 0.746 | 0.388 |
| Yes | 36 (37.1) | 18 (41.9) | 18 (33.3) |  |  |
| No | 61 (62.9) | 25 (58.1) | 36 (66.7) |  |  |
| Parasomnia |  |  |  | 0.347 | 0.556 |
| Yes | 50 (46.7) | 23 (50.0) | 27 (44.3) |  |  |
| No | 57 (53.3) | 23 (50.0) | 34 (55.7) |  |  |

| Age of onset (years) | 2.77 (13.2) | 26.3 (11.6) | 28.7 (14.3) | 0.938 | 0.351 |
| --- | --- | --- | --- | --- | --- |
| Age of first hospitalization | 30.5 (15.8) | 29.0 (15.1) | 31.5 (16.3) | 0.514 | 0.61 |
| Number of major depressive episodes | 3.0 (2.9) | 2.6 (2.2) | 3.2 (3.3) | 1.138 | 0.258 |
| Number of hospitalization | 1.1 (1.9) | 0.9 (1.8) | 1.3 (2.0) | 1.046 | 0.298 |
| Suicide attempt |  |  |  | 0.059 | 0.809 |
| Yes | 27 (25.5) | 12 (26.7) | 15 (24.6) |  |  |
| No | 79 (74.5) | 33 (73.3) | 46 (75.4) |  |  |
| Suicidal ideation |  |  |  | < 0.001 | 0.982 |
| Yes | 65 (60.7) | 28 (60.9) | 37 (60.7) |  |  |
| No | 42 (39.3) | 18 (39.1) | 24 (39.3) |  |  |
| Family history of depression |  |  |  | 1.628 | 0.202 |
| Yes | 60 (61.2) | 30 (68.2) | 30 (55.6) |  |  |
| No | 38 (38.8) | 14 (31.8) | 24 (44.4) |  |  |
| Family history of suicide |  |  |  | 1.988 | 0.159 |
| Yes | 21 (20.8) | 12 (27.3) | 9 (15.8) |  |  |
| No | 80 (79.2) | 32 (72.7) | 48 (84.2) |  |  |

| Sad mood |  |  |  | 0.83 | 0.362 |
| --- | --- | --- | --- | --- | --- |
| Yes | 85 (81.7) | 35 (77.8) | 50 (84.7) |  |  |
| No | 19 (18.3) | 10 (22.2) | 9 (15.3) |  |  |
| Loss of interest |  |  |  | 0.103 | 0.748 |
| Yes | 90 (85.7) | 40 (87.0) | 50 (84.7) |  |  |
| No | 15 (14.3) | 6 (13.0) | 9 (15.3) |  |  |
| Weight loss |  |  |  | 0.778 | 0.378 |
| Yes | 30 (30.9) | 11 (26.2) | 19 (34.5) |  |  |
| No | 67 (69.1) | 31 (73.8) | 36 (65.5) |  |  |
| Weight gain |  |  |  | 8.223 | **0.004** |
| Yes | 33 (33.3) | 21 (48.8) | 12 (21.4) |  |  |
| No | 66 (66.7) | 22 (51.2) | 44 (78.6) |  |  |
| Hypersomnia |  |  |  | 11.523 | **< 0.001** |
| Yes | 14 (13.3) | 12 (26.1) | 2 (3.4) |  |  |
| No | 91 (86.7) | 34 (73.9) | 57 (96.6) |  |  |
| Insomnia |  |  |  | 2.089 | 0.148 |
| Yes | 96 (91.4) | 40 (87.0) | 56 (94.9) |  |  |
| No | 9 (8.6) | 6 (13.0) | 3 (5.1) |  |  |
| Psychomotor retardation |  |  |  | 0.27 | 0.603 |
| Yes | 51 (54.3) | 21 (51.2) | 30 (56.6) |  |  |
| No | 43 (45.7) | 20 (48.8) | 23 (43.4) |  |  |
| Fatigue |  |  |  | 0.558 | 0.455 |
| Yes | 98 (96.1) | 43 (97.7) | 55 (94.8) |  |  |
| No | 4 (3.9) | 1 (2.3) | 3 (5.2) |  |  |
| Feelings of guilt |  |  |  | 0.82 | 0.365 |
| Yes | 74 (77.1) | 35 (81.4) | 39 (73.6) |  |  |
| No | 22 (22.9) | 8 (18.6) | 14 (26.4) |  |  |
| Difficulty concentrating |  |  |  | 0.24 | 0.625 |
| Yes | 87( 87.9) | 37 (86.0) | 50 (89.3) |  |  |
| No | 12 (12.1) | 6 (14.0) | 6 (10.7) |  |  |
| Seasonality |  |  |  | 0.496 | 0.481 |
| Yes | 26 (24.1) | 10 (20.8) | 16 (26.7) |  |  |
| No | 82 (75.9) | 38 (79.2) | 44 (73.3) |  |  |

UP = unipolar disorder; UP-N = unipolar disorder with nap; BMI = body mass index; PTSD = posttraumatic stress disorder.

Table S2. Demographic and clinical characteristics in the bipolar napping group (BD-N) compared to the bipolar non napping group (BD).

| Socio-demographic characteristics | Total sample  N = 66 | BD-N  N = 26 | BD  N = 40 | t-test ou **χ^2^** or Wilcoxon | *p-value* |
| --- | --- | --- | --- | --- | --- |
|  | Groups mean (SD) or n (%) | | |  |  |
| Age (years) | 43.8 (13.2) | 45.2 (12.7) | 42.7 (12.2) | 0.492 | 0.627 |
| Gender |  |  |  | 3.994 | **0.046** |
| Male | 25 (37.9) | 6 (23.1) | 19 (47.5) |  |  |
| Female | 41 (62.1) | 20 (76.9) | 21 (52.5) |  |  |
| Physical activity |  |  |  | 0.223 | 0.637 |
| Yes | 37 (67.3) | 12 (63.2) | 25 (69.4) |  |  |
| No | 18 (32.7) | 7 (36.8) | 11 (30.6) |  |  |
| Kids |  |  |  | 0.136 | 0.713 |
| Without | 40 (66.7) | 18 (69.2) | 22 (64.7) |  |  |
| With | 20 (33.3) | 8 (30.8) | 12 (35.3) |  |  |
| Marital status |  |  |  | 1.514 | 0.219 |
| Single | 37 (56.1) | 17 (65.4) | 20 (50.0) |  |  |
| Married | 29 (43.9) | 9 (34.6) | 20 (50.0) |  |  |
| Professional status |  |  |  | 2.864 | 0.091 |
| In activity | 43 (95.6) | 17 (89.5) | 26 |  |  |
| Without activity | 2 (4.4) | 2 (10.5) | 0 |  |  |
| BMI (kg/m2) | 25.2 (4.9) | 25.1 (5.3) | 25.2 (5.0) | 0.109 | 0.914 |
| Coffee consumption |  |  |  | 1.321 | 0.25 |
| Yes | 38 (69.1) | 15 (78.9) | 23 (63.9) |  |  |
| No | 17 (30.9) | 4 (21.1) | 13 (36.1) |  |  |
| Tea consumption |  |  |  | 0.73 | 0.393 |
| Yes | 15 (26.8) | 4 (20.0) | 11 (30.6) |  |  |
| No | 41 (73.2) | 16 (80.0) | 25 (69.4) |  |  |
| Smoking |  |  |  | 0.11 | 0.741 |
| Yes | 18 (34.0) | 7 (36.8) | 11 (32.4) |  |  |
| No | 35 (66.0) | 12 (63.2) | 23 (67.6) |  |  |
| Alcohol |  |  |  | 0 | 1 |
| Yes | 10 (15.4) | 4 (15.4) | 6 (15.4) |  |  |
| No | 55 (84.6) | 22 (84.6) | 33 (84.6) |  |  |
| Antidepressant treatments |  |  |  | 0.42 | 0.517 |
| Yes | 25 (39.1) | 11 (44.0) | 14 (35.9) |  |  |
| No | 39 (60.9) | 14 (56.0) | 25 (64.1) |  |  |
| Hypnotical treatments |  |  |  | 1.58 | 0.209 |
| Yes | 24 (37.5) | 7 (28.0) | 17 (43.6) |  |  |
| No | 40 (62.5) | 18 (72.0) | 22 (56.4) |  |  |
| Anxiolytic treatments |  |  |  | 0.301 | 0.583 |
| Yes | 28 (43.8) | 12 (48.0) | 16 (41.0) |  |  |
| No | 36 (56.3) | 13 (52.0) | 23 (59.0) |  |  |
| Antipsychotic treatments |  |  |  | 0.363 | 0.547 |
| Yes | 7 (10.9) | 2 (8.0) | 5 (12.8) |  |  |
| No | 57 (89.1) | 23 (92.0) | 34 (87.2) |  |  |
| Mood stabilizer treatments |  |  |  | 0.323 | 0.57 |
| Yes | 33 (51.6) | 14 (56.0) | 19 (48.7) |  |  |
| No | 31 (48.4) | 11 (44.0) | 20 (51.3) |  |  |
| Clinical characteristics | Total sample  N = 66 | Nap  N = 26 | No nap  N = 40 | t-test ou χ2 or Wilcoxon | p-value |
| Panic disorder |  |  |  | 1.833 | 0.176 |
| Yes | 16 (24.2) | 4 (15.4) | 12 (30.0) |  |  |
| No | 50 (75.8) | 22 (84.6) | 28 (70.0) |  |  |
| PTSD |  |  |  | 0.461 | 0.497 |
| Yes | 18 (27.7) | 6 (23.1) | 12 (30.8) |  |  |
| No | 47 (72.3) | 20 (76.9) | 27 (69.2) |  |  |
| Hyperarousal |  |  |  | 0.018 | 0.893 |
| Yes | 28 (47.5) | 10 (41.7) | 18 (51.4) |  |  |
| No | 31 (52.5) | 14 (58.3) | 17 (48.6) |  |  |
| Sleep inertia |  |  |  | 1.516 | 0.218 |
| Yes | 9 (15.3) | 5 (22.7) | 4 (10.8) |  |  |
| No | 50 (84.7) | 17 (77.3) | 33 (89.2) |  |  |
| Daytime sleepiness |  |  |  | 4.875 | 0.027 |
| Yes | 27 (45.0) | 14 (63.6) | 13 (34.2) |  |  |
| No | 33 (55.0) | 8 (36.4) | 25 (65.8) |  |  |
| Obstructive sleep apnea syndrome |  |  |  | 0.263 | 0.608 |
| Yes | 16 (28.6) | 6 (25.0) | 10 (31.3) |  |  |
| No | 40 (71.4) | 18 (75.0) | 22 (68.8) |  |  |
| Parasomnia |  |  |  | 1.34 | 0.247 |
| Yes | 18 (28.1) | 5 (20.0) | 13 (33.3) |  |  |
| No | 46 (71.9) | 20 (80.0) | 26 (66.7) |  |  |

| Age of onset (years) | 22.6 (9.9) | 23.5 (10.6) | 21.8 (9.9) | 0.501 | 0.62 |
| --- | --- | --- | --- | --- | --- |
| Age of first hospitalization | 38.3 (14.0) | 40.7 (13.1) | 36.5 (12.1) | 0.669 | 0.513 |
| Number of major depressive episodes | 6.4 (7.3) | 8.3 (6.6) | 4.8 (3.9) | 1.101 | 0.291 |
| Number of hospitalization | 3.7 (6.4) | 3.4 (4.8) | 3.8 (4.7) | 0.163 | 0.872 |
| Suicide attempt |  |  |  | < 0.001 | 0.977 |
| Yes | 15 (23.8) | 6 (24.0) | 9 (23.7) |  |  |
| No | 48 (76.2) | 19 (76.0) | 29 (76.3) |  |  |
| Suicidal ideation |  |  |  | 4.636 | **0.031** |
| Yes | 31 (49.2) | 17 (65.4) | 14 (37.8) |  |  |
| No | 32 (50.8) | 9 (34.6) | 23 (62.2) |  |  |
| Family history of depression |  |  |  | 2.439 | 0.118 |
| Yes | 33 (55.0) | 15 (68.2) | 18 (47.4) |  |  |
| No | 27 (45.0) | 7 (31.8) | 20 (52.6) |  |  |
| Family history of suicide |  |  |  | 0.77 | 0.38 |
| Yes | 10 (16.4) | 5 (21.7) | 5 (13.2) |  |  |
| No | 51 (83.6) | 18 (78.3) | 33 (86.8) |  |  |
| Clinical characteristics | Total sample  N = 66 | Nap  N = 26 | No nap  N = 40 | t-test ou **χ^2^** or Wilcoxon | *p-value* |
| Sad mood |  |  |  | 5.504 | **0.019** |
| Yes | 35 (55.6) | 19 (73.1) | 16 (43.2) |  |  |
| No | 28 (44.4) | 7 (26.9) | 21 (56.8) |  |  |
| Loss of interest |  |  |  | 2.222 | 0.136 |
| Yes | 35 (59.3) | 17 (70.8) | 18 (51.4) |  |  |
| No | 24 (40.7) | 7 (29.2) | 17 (48.6) |  |  |
| Weight loss |  |  |  | 2.302 | 0.129 |
| Yes | 10 (18.5) | 6 (28.6) | 4 (12.1) |  |  |
| No | 44 (81.5) | 15 (71.4) | 29 (87.9) |  |  |
| Weight gain |  |  |  | 0.307 | 0.579 |
| Yes | 13 (24.5) | 6 (28.6) | 7 (21.9) |  |  |
| No | 40 (75.5) | 15 (71.4) | 25 (78.1) |  |  |
| Hypersomnia |  |  |  | 4.232 | **0.04** |
| Yes | 16 (25.0) | 10 (38.5) | 6 (15.8) |  |  |
| No | 48 (75.0) | 16 (61.5) | 32 (84.2) |  |  |
| Insomnia |  |  |  | 1.069 | 0.301 |
| Yes | 50 (79.4) | 19 (73.1) | 31 (83.8) |  |  |
| No | 13 (20.6) | 7 (26.9) | 6 (16.2) |  |  |
| Psychomotor retardation |  |  |  | 0.712 | 0.399 |
| Yes | 29 (48.3) | 10 (41.7) | 19 (52.8) |  |  |
| No | 31 (51.7) | 14 (58.3) | 17 (47.2) |  |  |
| Fatigue |  |  |  | 3.782 | 0.052 |
| Yes | 55 (85.9) | 25 (96.2) | 30 (78.9) |  |  |
| No | 9 (14.1) | 1 (3.8) | 8 (21.1) |  |  |
| Feelings of guilt |  |  |  | 3.882 | **0.049** |
| Yes | 26 (44.8) | 13 (61.9) | 13 (35.1) |  |  |
| No | 32 (55.2) | 8 (38.1) | 24 (64.9) |  |  |
| Difficulty concentrating |  |  |  | 0.441 | 0.507 |
| Yes | 42 (68.9) | 17 (73.9) | 25 (65.8) |  |  |
| No | 19 (31.1) | 6 (26.1) | 13 (34.2) |  |  |
| Seasonality |  |  |  | 0.662 | 0.416 |
| Yes | 11 (17.5) | 3 (12.5) | 8 (20.5) |  |  |
| No | 52 (82.5) | 21 (87.5) | 31 (79.5) |  |  |

BD = bipolar disorder; BD-N = bipolar disorder with nap; BMI = body mass index; PTSD = posttraumatic stress disorder.

Table S3. Subjective sleep and circadian rhythm markers assessed with self-report questionnaires in the unipolar napping group (UD-N) compared to the unipolar non napping group (UD).

|  | Total sample  N = 107 | UD-N  N = 46 | UD  N = 61 | t-test or Wilcoxon | *p-value* |
| --- | --- | --- | --- | --- | --- |
|  | Groups mean (SD) or n (%) | | |  |  |
| Depressive symptoms (MADRS) | 24.84 (8.64) | 23.73 (8.89) | 25.00 (8.43) | 0.683 | 0.497 |
| Sleep disturbances (PSQI total score) | 12.81 (3.87) | 12.14 (3.57) | 13.44 (4.09) | 1.659 | 0.105 |
| PSQI-sleep subjective quality | 2.31 (0.66) | 2.32 (0.69) | 2.33 (0.64) | 0.074 | 0.941 |
| PSQI-sleep latency | 2.06 (1.05) | 1.95 (1.05) | 2.15 (1.04) | 0.9 | 0.371 |
| PSQI-sleep duration | 1.14 (1.19) | 0.85 (1.13) | 1.33 (1.19) | 1.987 | **0.049** |
| PSQI-sleep efficiency | 1.51 (1.21) | 1.20 (1.19) | 1.76 (1.19) | 2.321 | **0.023** |
| PSQI-sleep disturbance | 1.83 (0.61) | 1.88 (0.64) | 1.80 (0.59) | 0.611 | 0.543 |
| PSQI-sleep promoting medication | 2.12 (1.25) | 2.00 (1.34) | 2.25 (1.16) | 0.974 | 0.333 |
| PSQI-sleep dysfunction | 1.78 (0.76) | 1.80 (0.87) | 1.82 (0.64) | 0.082 | 0.935 |
| Seasonality (GSS) | 9.96 (6.32) | 11.53 (6.36) | 9.09 (6.35) | 1.907 | 0.06 |
| Epworth Spleepiness Scale (ESS) | 8.70 (5.52) | 11.41 (5.15) | 6.72 (4.46) | 4.806 | **< 0.001** |
| Insomnia symptoms (ISI total score) | 18.18 (6.20) | 18.21 (5.38) | 18.64 (6.40) | 0.379 | 0.705 |
| ISI 1A (Difficulty Falling Asleep) | 2.45 (1.20) | 2.13 (1.22) | 2.70 (1.18) | 2.374 | **0.02** |
| ISI 1B (Difficulty Maintaining Sleep) | 2.52 (1.18) | 2.44 (1.24) | 2.64 (1.10) | 0.826 | 0.411 |
| ISI 1C (Early Morning Awakening) | 2.56 (1.33) | 2.54 (1.33) | 2.61 (1.29) | 0.239 | 0.812 |
| ISI 2 (Dissatisfaction with sleep pattern) | 3.29 (0.81) | 3.29 (0.76) | 3.26 (0.89) | 0.186 | 0.853 |
| ISI 3 (Interference with daily functioning) | 3.20 (0.73) | 3.24 (0.65) | 3.16 (0.83) | 0.613 | 0.541 |
| ISI 4 (Noticeability due to sleep issues) | 2.44 (1.05) | 2.27 (0.79) | 2.54 (1.17) | 1.391 | 0.167 |
| ISI 5 (Distress caused by sleep issues) | 2.84 (0.91) | 2.73 (0.89) | 2.93 (0.96) | 1.069 | 0.288 |
| Chronotype (Horne et Otsberg questionnaire) | 46.17 (11.22) | 44.77 (10.26) | 46.87 (11.48) | 0.958 | 0.341 |
| Hypersomnia Severity Index total | 17.77 (5.56) | 20.00 (4.81) | 15.71 (5.84) | 3.37 | **0.001** |
| HSI 1A - Sleep duration | 0.88 (1.28) | 1.13 (1.50) | 0.65 (1.03) | 1.515 | 0.136 |
| HSI 1B - Sleep inertia | 2.35 (1.30) | 2.72 (1.14) | 2.03 (1.38) | 2.292 | **0.025** |
| HSI 1C - Sleep during day | 1.36 (1.31) | 1.97 (1.23) | 0.71 (1.11) | 4.45 | **< 0.001** |
| HSI 1D - Sleepiness | 1.94 (1.24) | 2.22 (0.97) | 1.66 (1.44) | 1.933 | **0.058** |
| HSI 2 - Satisfaction | 2.66 (1.39) | 2.84 (1.27) | 2.58 (1.48) | 0.804 | 0.424 |
| HSI 3 - Daytime dysfunction | 3.06 (0.92) | 3.28 (0.58) | 2.89 (1.11) | 1.865 | **0.067** |
| HSI 4 - Symptoms apparentness | 2.25 (1.12) | 2.38 (0.94) | 2.19 (1.27) | 0.697 | 0.488 |
| HSI 5 - Distress | 2.84 (0.86) | 2.75 (0.76) | 2.89 (0.95) | 0.706 | 0.483 |
| HSI 6 - Sleep attacks | 0.45 (0.90) | 0.72 (1.02) | 0.18 (0.65) | 2.551 | **0.014** |

MADRS = Montgomery-Asberg Depression Rating Scale; PSQI = Pittsburgh Sleep Quality Index. GSS = General Seasonality Score; ESS = Epworth Sleepiness Scale; ISI = Insomnia Severity Index; HSI = Hypersomnia Severity Index

Table S4. Subjective sleep and circadian rhythm markers assessed with self-report questionnaires in the bipolar napping group (BD-N) compared to the bipolar non napping group (BD).

|  | Total sample  N = 66 | BD-N N = 26 | BD  N = 40 | t-test ou **χ^2^** or Wilcoxon | *p-value* |
| --- | --- | --- | --- | --- | --- |
|  | Groups mean (SD) or n (%) | | |  |  |
| MADRS | 22.79 (9.61) | 21.20 (8.40) | 23.11 (9.17) | 0.562 | 0.58 |
| PSQI total score | 10.21 (4.44) | 8.80 (3.26) | 11.42 (5.01) | 1.697 | 0.102 |
| PSQI-sleep subjective quality | 1.86 (1.03) | 1.80 (1.14) | 2.16 (0.90) | 0.865 | 0.401 |
| PSQI-sleep latence | 1.40 (1.01) | 0.80 (0.63) | 1.68 (1.00) | 2.9 | **0.008** |
| PSQI-sleep duration | 0.79 (1.09) | 0.50 (0.71) | 1.16 (1.26) | 1.801 | 0.083 |
| PSQI-sleep efficiency | 0.79 (1.07) | 0.40 (0.97) | 1.11 (1.15) | 1.747 | 0.095 |
| PSQI-sleep disturbance | 1.43 (0.65) | 1.50 (0.53) | 1.58 (0.69) | 0.343 | 0.735 |
| PSQI-sleep promoting medication | 1.89 (1.37) | 1.90 (1.37) | 2.00 (1.33) | 0.189 | 0.853 |
| PSQI-sleep dysfunction | 1.80 (0.93) | 1.90 (0.88) | 1.74 (0.93) | 0.466 | 0.646 |
| Seasonality (GSS) | 8.63 (6.04) | 11.38 (5.11) | 8.25 (5.95) | 1.612 | 0.118 |
| Epworth Spleepiness Scale (ESS) | 9.42 (5.84) | 11.85 (5.34) | 8.25 (5.88) | 1.816 | 0.08 |
| ISI total score | 11.18 (9.74) | 11.95 (8.93) | 12.98 (9.5) | 0.348 | 0.73 |
| ISI 1A (Difficulty Falling Asleep) | 1.97 (1.48) | 1.14 (1.17) | 2.64 (1.34) | 3.316 | **0.004** |
| ISI 1B (Difficulty Maintaining Sleep) | 1.72 (1.41) | 1.21 (1.42) | 2.07 (1.21) | 1.718 | 0.098 |
| ISI 1C (Early Morning Awakening) | 2.17 (1.56) | 2.07 (1.73) | 2.14 (1.40) | 0.12 | 0.906 |
| ISI 2 (Dissatisfaction with sleep pattern) | 3.05 (1.13) | 3.00 (1.11) | 3.03 (1.20) | 0.078 | 0.939 |
| ISI 3 (Interference with daily functioning) | 2.93 (1.14) | 3.00 (0.96) | 2.80 (1.32) | 0.469 | 0.643 |
| ISI 4 (Noticeability due to sleep issues) | 2.72 (1.22) | 2.86 (1.17) | 2.50 (1.27) | 0.79 | 0.437 |
| ISI 5 (Distress caused by sleep issues) | 2.78 (1.20) | 2.93 (1.33) | 2.57 (1.08) | 0.801 | 0.431 |
| Horne et Otsberg (MEQ) | 47.52 (14.14) | 46.82 (15.24) | 46.73 (13.32) | 0.015 | 0.988 |
| Hypersomnia Severity Index total | 17.77 (7.35) | 21.22 (5.61) | 15.08 (7.91) | 2.08 | 0.051 |
| HSI 1A - Sleep duration | 1.14 (1.49) | 1.44 (1.67) | 1.00 (1.41) | 0.645 | 0.529 |
| HSI 1B - Sleep inertia | 1.95 (1.36) | 2.56 (1.24) | 1.50 (1.38) | 1.841 | 0.082 |
| HSI 1C - Sleep during day | 1.82 (1.50) | 2.56 (1.42) | 1.17 (1.34) | 2.27 | **0.037** |
| HSI 1D - Sleepiness | 1.77 (1.02) | 2.11 (1.05) | 1.67 (0.89) | 1.022 | 0.322 |
| HSI 2 - Satisfaction | 2.59 (1.53) | 2.89 (1.27) | 2.25 (1.71) | 0.982 | 0.339 |
| HSI 3 - Daytime dysfunction | 2.82 (1.30) | 3.44 (0.73) | 2.25 (1.42) | 2.506 | **0.023** |
| HSI 4 - Symptoms apparentness | 2.55 (1.18) | 2.78 (1.20) | 2.42 (1.24) | 0.672 | 0.51 |
| HSI 5 - Distress | 2.73 (1.28) | 3.00 (1.32) | 2.42 (1.24) | 1.027 | 0.319 |
| HSI 6 - Sleep attacks | 0.43 (0.87) | 0.44 (1.01) | 0.45 (0.82) | 0.024 | 0.981 |

MADRS = Montgomery-Asberg Depression Rating Scale; PSQI = Pittsburgh Sleep Quality Index. GSS = General Seasonality Score; ESS = Epworth Sleepiness Scale; ISI = Insomnia Severity Index; HSI = Hypersomnia Severity Index
